# Prevalence and gender disparity of those who screen positive for depression in China by the classification of the employer and industry: a cross-sectional, population-based study

**DOI:** 10.1186/s12888-023-04557-7

**Published:** 2023-01-24

**Authors:** Shanquan Chen, Yuqi Wang, Rui She

**Affiliations:** 1 Department of Psychiatry, University of Cambridge, Cambridge, CB2 0SZ UK; 2 Department of Computer Science, University College London, London, WC1E 6BT UK; 3 Department of Rehabilitation Sciences, The Hong Kong Polytechnic University, Hong Kong, China

**Keywords:** Depression, Prevalence, Gender disparity, Industrial classification, Employer classification, China

## Abstract

**Background:**

The important role of mental health in sustainable economic development is gradually being recognized. This study aimed to evaluate the prevalence and gender disparity of those who screen positive for depression in China by the employer and industrial classification.

**Methods:**

We used data from a nationally representative survey, the China Family Panel Studies. Depression was judged by the Centre for Epidemiologic Studies Depression Scale. Employer classifications were categorized according to the local characteristics of Mainland China. Industrial classifications were defined using level-1 of the China version of the International Standard Industrial Classification of All Economic Activities. Weighted logistic regressions were fitted to estimate the gender disparities, controlling for confounders.

**Results:**

Forty eight thousand six hundred twenty eight adults were included. 18.7% (95%CI 18.1–19.4) of sampled adults were screened positive for depression symptoms, with 16.6% (95%CI 15.8–17.5) in males vs 21.0% (95%CI 20.1–22.0) in females. By classification of the employer, the prevalence was lowest among those employed by Government/party organisations (11.8%, 95%CI 8.9–15.4), and highest in those self-employed (21.8%, 95%CI 20.8–22.9); the gender disparity was mainly found in those employed by Sole proprietorship (Adjusted odds ratio [AOR] = 1.95, 95%CI 1.19–3.19) and Private enterprise (AOR = 1.34, 95%CI 1.13–1.59), as well as those self-employed (AOR = 1.49, 95%CI 1.3–1.17). By industrial classification, the prevalence was lowest among those who worked in the industry of Real estate (7.2%, 95%CI 4.8–10.6), and highest among those who worked in the industry of Agriculture, forestry, animal husbandry and fishing (22.9%, 95%CI 15.5–32.4); the gender disparity was mainly found in those who worked in the industry of Agriculture, forestry, animal husbandry and fishing (AOR = 3.29, 95%CI 1.18–9.15), Manufacturing (AOR = 1.41, 95% CI 1.09–1.82), Wholesale and retail trade (AOR = 1.48, 95% CI 1.07–2.06), and Accommodation and food service (AOR = 1.91, 95% CI 1.15–3.18).

**Conclusion:**

The prevalence of depression in China had a wide variation by classifications of the employer and industry. Gender disparities were identified among workers from Sole proprietorship, Private enterprise, and self-employed, or workers from the industry of Agriculture, forestry, animal husbandry and fishing, Manufacturing, Wholesale and retail trade, and Accommodation and food service.

## Background

A growing body of evidence emphasizes the importance of mental health in sustainable economic development. It is estimated that between 2010 and 2030, mental health will become the main cause of chronic diseases and will cause a loss of US$16 trillion to the global economy, accounting for about one-third of all costs of chronic diseases [1]. In China, the economy had rapid development at annual rates of over 6% and often even higher than 10% in the past three decades [2]. During this period, China made remarkable improvements in the treatment and control of physical diseases, but the process of improving mental health has been left far behind [3]. From 2010 to 2019, discharges because of mental illness increased by approximately a rate of 12.6% per year, which reached 3.3 million in 2019; outpatient visits increased similarly, by 10.3% per year, which reached 60 million visits in 2019 [4]. It was estimated that in 2013 the total annual costs of mental disorders in China accounted for more than 15% of its total health expenditure [5]. As of today, the burden due to mental disorders could be higher. The focus on the mental problem is essential for China to reduce the negative influence of mental problems on its even global sustainable economic development.

In China, about 15.9–38.6% of the general population suffered from common mental health problems [6–10]. Previous studies have also estimated the prevalence of mental disorders by age, gender, and year [10–14]. However, the evidence from the workplace is few, and available evidence can only be seen in concerted groups of people, such as healthcare workers, migrant workers, and those employed in the entertainment sector [15–21]. Depression is one of the common types of mental disorders and a leading cause of disability [22], yet evidence of depression in the workplace is rarer. Nevertheless, the available evidence already alarms a worrying situation. A cross-sectional survey based on 807 migrant factory workers in China indicated that 60.3% of the respondents had mild-to-severe depression [23]. A cross-sectional survey based on 4520 physicians from 41 tertiary psychiatric hospitals in China indicated that 44.9% of physicians had depression [24]. A cross-sectional survey based on 358 female migrants from entertainment venues in China indicated that 31.0% of respondents had clinically significant depressive symptoms [17]. A cross-sectional survey based on 1500 university teachers in China indicated that 58.9% of university teachers had depressive symptoms [25]. By contrast, a recent meta-analysis based on 218 studies estimated that the overall prevalence of depression among migrant workers was 28.6% [26], and a study from South Korea based on 3190 female wage workers indicated that 20.7% of the participants had depressive symptoms [27].

People are exposed to their unique occupational environments depending on the kind of employers or the type of industries they work for. For instance, the traditional belief in China is that working in government departments is more stable than working in enterprises despite their higher income; and the construction industry has always been male-dominated, while the sales and service industry tends to be female-dominated. Understanding mental health by classification of the employer and industry could be beneficial to the individualization and pertinence of policies or intervention measures. However, in China, no study was conducted on mental health by the classification of the employer and industry in a systematic way.

It was documented that females are more likely to have mental health problems than males [28], while studies from China involving workplace-participant found inconsistent evidence and indicated that there was no gender disparity [23, 24] or the prevalence was also significantly higher in females [7, 8, 25]. This difference may be due to the fact that participants come from different industries where there may be different intersections between gender parenting, socialization, and roles [12]. However, little is known about how these gender disparities vary over the industries. Corresponding evidence is necessary to proactively plan equal and sustainable healthcare frameworks, especially in the context that China has been experiencing rapid and uneven progress in gender equity development (like more women participated in the labour market and even in some areas women have begun to outperform men [12]).

This study aimed to evaluate the prevalence and gender disparity of those who screen positive for depression in China by the classification of the employer and industry.

## Methods

### Database and participants

We used data from the China Family Panel Studies (CFPS). The CFPS is a general-purpose, nationally representative, and longitudinal survey conducted by the Institute of Social Science Survey of Peking University on people aged 9 or above. The survey sample was drawn from 31 provinces/cities/autonomous regions of China using a multistage probability proportional to size sampling method, representing 95% of the Chinese population. The survey has been conducted every 2 years since the baseline survey in 2010. In each wave, about 30,000 individuals from 15,000 families were interviewed. Individuals were interviewed using computer-assisted personal interviewing (CAPI) technology, provided by the Survey Research Center (SRC) at the University of Michigan, with an approximate response rate of 79%. Information collected by CFPS included socio-economy, demography, family dynamics and relationships, and physical and psychological health. Detailed descriptions of CFPS, such as the sampling method and quality-control procedures, can be found elsewhere [29, 30].

This study used surveys from the 2016 wave and the 2018 wave since the instrument used to screen for depression in these two waves was validated (see following part of measures) but not in other waves. The inclusion criteria were those aged between 19 and 55 for females or between 19 and 60 for males, taking into account the legal working age and retirement age by gender in China.

### Measures

#### Depression

Depression was judged by the Centre for Epidemiologic Studies Depression (CES-D) Scale. This scale was developed for use in studies of the epidemiology of depressive symptomatology in the general population [31]. Its purpose differs from previous depression scales that have been used mainly for diagnosis at clinical intake and/or evaluation of the severity of illness over the course of treatment [31]. Originally, CES-D is a 20-item scale that asks individuals to rate how often over the past week they experienced symptoms associated with depression, such as restless sleep, poor appetite, and feeling lonely. Response options range from 0 to 3 for each item (0 = Rarely or None of the Time, 1 = Some or Little of the Time, 2 = Moderately or Much of the time, 3 = Most or Almost All the Time). The CES-D has kinds of short versions. CFPS used its full-item version and 8-item version in 2016 (CES-D-20) and 2018 (CES-D-8), respectively. The total score of CES-D-8 ranges from 0 to 24 with a validated cut-off point of 9 for probable depression, and the total score of CES-D-20 ranges from 0 to 60 with a validated cut-off point of 16 for probable depression [31, 32].

#### Classification of the employer and industry

The employer was grouped into the following categories according to the local characteristics of Mainland China, including Government/party organization, State-owned/collectively-owned public institution, State-owned/state-controlled enterprise, Private enterprise, Enterprise invested by foreign/Hong Kong, Macao and Taiwan, Sole proprietorship, Private non-enterprise organization/association/foundation, and others. To maximise the coverage and the comparability of this classification, we also included non-employed and self-employed into the classification of the employer.

Industrial classifications were defined using level-1 of the China version of the International Standard Industrial Classification of All Economic Activities (ISIC), including industries such as Agriculture, forestry, fishing, Mining and quarrying, Manufacturing, Construction, etc. [33].

#### Other variables

Socio-demographic characteristics included age (years), gender (male vs female), marital status (married/cohabitation, never married, and widowed/divorced/separated), education attained (illiterate, primary school, middle school, high school or equivalent, and bachelor or above), and average annual household income per person.

We also investigated the following variables because of their identified influence on depression in China, including residence place (urban vs rural), self-rated health status (poor or lower, fair, and good or above), and survey year (2016 or 2018) [13, 34, 35].

### Statistical analysis

Data from 2016 and 2018 were pooled for analysis. In the descriptive analyses, we reported categorical variables as numbers (percentage), and continuous variables as mean (standard deviation, SD).

We estimated the prevalence of depression and its 95% confidence interval (CI) by the classification of the employer and industry, as well as by gender. Survey weights were used to account for the complex survey design.

To estimate the gender difference, we fitted weighted logistic regression models, with depression (yes or no) as the dependent variable and gender (with males as the reference) as the key predictor, controlling for age, marital status, education attained, income level, residence place, self-rated health status, and survey year.

Analyses used R version 3.6.0. *P* < 0.05 was considered statistically significant. Results are reported following the STROBE checklist for cohort studies.

## Results

In this study, 48,628 adults were included with a mean (SD) age of 38.9 (11.4). Among them, about half were females (47.3%), 80.0% were married or in cohabitation, 33.1% attained an education degree of high school or above, 50.6% lived in rural areas, and 38.5% reported good or above health status. The average annual household income per person was 31,501.5 (SD 57,746.1) RMB or about 4713.8 (SD 8,596.1) dollars in the average exchange rate during 2016 and 2018. 86.9% of respondents were occupied, out of which 49.8% were employed and 37.1% were self-employed. By classification of the employer (only for those employed), 30.3% were employed in Private enterprise, followed by State-owned/state-controlled enterprise (5.9%), State-owned/collectively-owned public institution (3.8%), and Sole proprietorship (3.2%). By industrial classification (for both employed and self-employed), 15.8% worked in the industry of Manufacturing, followed by Wholesale and retail (8.7%), Construction (6.8%), and Hotel and catering service (4.6%). Details of the basic characteristics were presented in Table 1.

**Table 1:** Basic description

**Characteristic**	**No. (%) of participants**
**Gender (= Female)**	22,985 (47.3)
**Age**	38.9 (11.4)
**Residence place**	
Rural	24,622 (50.6)
Urban	24,006 (49.4)
**Self-rated health status**	
Poor or lower	12,100 (24.9)
Fair	19,095 (39.3)
Good or above	17,433 (35.8)
**Education attained**	
Illiterate	7297 (15.0)
Primary school	9725 (20.0)
Middle school	15,504 (31.9)
High school or equivalent	8787 (18.1)
Bachelor or above	7315 (15.0)
**Marital Status**	
Married/Cohabitation	38,925 (80.0)
Never married	7901 (16.2)
Widowed/divorced/separated	1802 (3.7)
**Total annual household income before taxes per person**	31,501.5 (57,446.1)
**Depression present (= yes)**	9587 (19.7)
**Classification of employer**	
Employed	24,220 (49.8)
Enterprise invested in by foreign/Hong Kong, Macao and Taiwan	745 (1.5)
Government/Party organization	1169 (2.4)
Sole proprietorship	1535 (3.2)
Private enterprise	14,743 (30.3)
Private non-enterprise organization/association/guild/foundation	295 (0.6)
State-owned/Collectively-owned public institutions	1830 (3.8)
State-owned/State-controlled enterprise	2859 (5.9)
Others	1044 (2.1)
Self-employed	18,032 (37.1)
Non-employed	6376 (13.1)
**Industrial classifications**	
Agriculture, forestry, animal husbandry and fishing	688 (1.4)
Construction	3302 (6.8)
Cultural, physical and entertainment	451 (0.9)
Education	1616 (3.3)
Financial and insurance	587 (1.2)
Sanitation, social security and social welfare	762 (1.6)
Accommodation and food service	2257 (4.6)
Information transfer, computer service and software	496 (1.0)
Manufacturing	7706 (15.8)
Mining and quarrying	451 (0.9)
Others	14,748 (30.3)
Production and supply of electricity power, gas, and water	410 (0.8)
Public administration and social organisation	1176 (2.4)
Real estate	523 (1.1)
Leasehold and business service	657 (1.4)
Neighbourhood services and other service	1369 (2.8)
Scientific research, technical service and geologic examination	129 (0.3)
Traffic, storage, and mail business	1643 (3.4)
Water conservancy, environment and public institution management	259 (0.5)
Wholesale and retail trade	4224 (8.7)

After considering the survey weight, 18.7% (95%CI 18.1–19.4) of sampled adults were screened positive for depression symptoms, with 16.6% (95% CI 15.8–17.5) in males vs 21.0% (95% CI 20.1–22.0) in females. After controlling for age, marital status, education attained, income level, residence place, self-rated health status, and survey year, females had a 1.38-time likelihood of being screened positive for depression symptoms than males (adjusted Odds ratio[AOR] = 1.38, 95%CI 1.26–1.51).

**Fig. 1:**
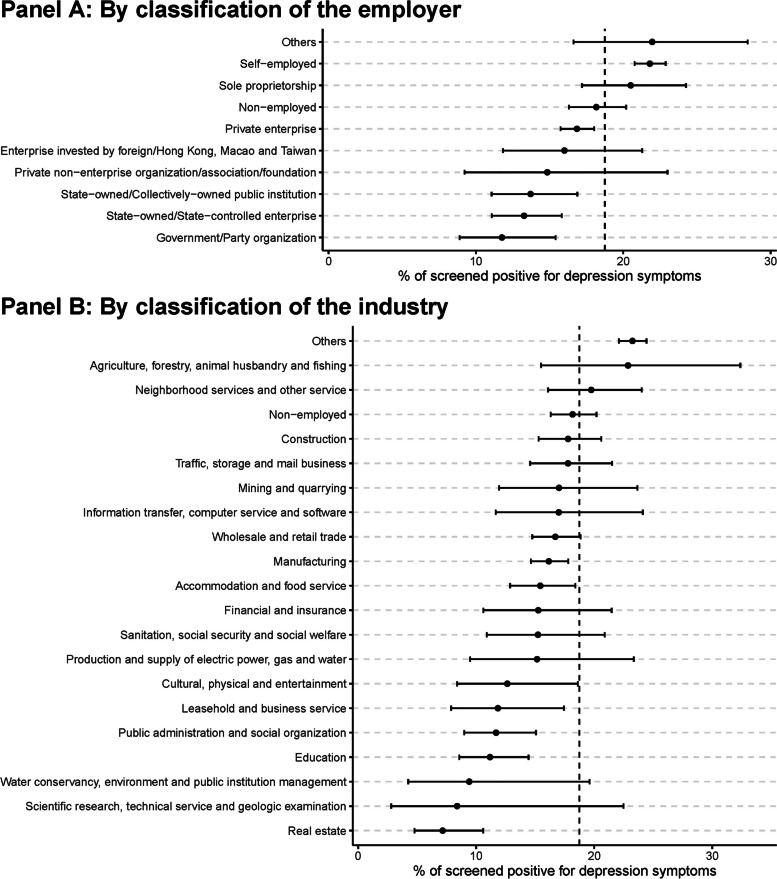
Prevalence of those who screen positive for depression in China, by classification of the employer and industry. Shown is the prevalence and its 95% confidence interval (CI) of populations who screen positive for depression

Figure 1 showed the prevalence of depression by classification of the employer (Panel A) and industry (Panel B). Panel A indicated that the prevalence was lowest among those employed by Government/party organizations (11.8%, 95%CI 8.9–15.4), followed by State-owned/state-controlled enterprises (13.3%, 95%CI 11.1–15.8), State-owned/collectively-owned public institutions (13.7%, 95%CI 11.1–16.9), Private non-enterprise organizations/associations/foundations (14.8%, 95%CI 9.2–23), Enterprise invested by foreign/Hong Kong, Macao and Taiwan(16.0%, 95%CI 11.8–21.3), Private enterprise (16.9%, 95%CI 15.7–18.0), Non-employed (18.2%, 95%CI 16.3–20.2), Sole proprietorship(20.5%, 95%CI 17.2–24.3), and Self-employed (21.8%, 95%CI 20.8–22.9). Panel B indicated that the prevalence was lowest among those who worked in the industry of Real estate (7.2%, 95%CI 4.8–10.6), followed by Scientific research, technical service and geologic examination (8.4%, 95%CI 2.8–22.5), Water conservancy, environment and public institution management (9.4%, 95%CI 4.2–19.6), Education (11.2%, 95%CI 8.6–14.4), and Public administration and social organization (11.7%, 95%CI 9.0–15.1), while the prevalence was highest among those working in the industry of Agriculture, forestry, animal husbandry and fishing (22.9%, 95%CI 15.5–32.4), followed by Neighborhood service and other services (19.8%, 95%CI 16.1–24.0), Non-employed (18.2%, 95%CI 16.3–20.2), and Construction (17.8%, 95%CI 15.3–20.6), and Traffic, storage and mail business (17.8%, 95%CI 14.6–21.5).

**Fig. 2:**
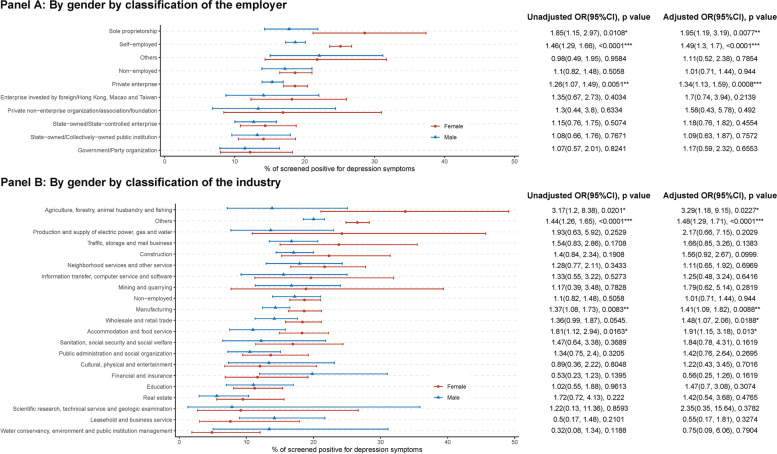
Gender disparity on the prevalence of those who screen positive for depression in China, by classification of the employer and industry. Shown are the prevalence and its 95% confidence interval (CI) of populations who screen positive for depression. The odds ratio (OR) and its 95CI% were estimated from weighted logistic with depression (yes or no) as the dependent variable and gender (with males as the reference) as the key predictor. Adjusted OR controlled for age, marital status, education attained, income level, residence place, self-rated health status, and survey year. * < 0.05, ** < 0.01, *** < 0.001

Figure 2 showed the gender disparity of the prevalence of depression by classification of the employer (Panel A) and industry (Panel B), and indicated that more females were screened positive for depression than males among those who worked under some types of employers or industries. Panel A indicated that the female-higher prevalence mainly happened in Sole proprietorship (AOR = 1.95, 95% CI 1.19–3.19), Self-employed (AOR = 1.49, 95% CI 1.3–1.17), and Private enterprise (AOR = 1.34, 95% CI 1.13–1.59). Panel B indicated that the female-higher prevalence mainly happened to those who worked in the industry of Agriculture, forestry, animal husbandry and fishing (AOR = 3.29, 95% CI 1.18–9.15), Manufacturing (AOR = 1.41, 95% CI 1.09–1.82), Wholesale and retail trade (AOR = 1.48, 95% CI 1.07–2.06), and Accommodation and food service (AOR = 1.91, 95% CI 1.15–3.18).

## Discussion

To the best of our knowledge, we firstly evaluated the prevalence of those who screen positive for depression in China by the employer and industrial classification, as well as corresponding gender disparities within each classification.

The prevalence of those who screen positive for depression in China (18.7%) was higher than that in India (7.6% among middle-aged adults) [36], Peru (about 7.1% among general populations) [37], Brazil (about 10% among general populations) [38, 39], the US (about 7% among those age 35–49) [40], Korean (6.1% among adults) [41], and Canadian (5.4% among employed working-age adults) [42], while lower than that in Ghana (25.2% among adults) [43] and Indonesia (23.5% among adults) [44]. Differently, the gender difference in the above prevalence in China (females had a 1.38-time likelihood of being screened positive for depression symptoms than males) was narrower than that in Peru (2.83) [37], Brazil (2.75) [38], and Korean (3.11) [41], while wider than that in Ghana (1.09) [43]. These differences may be related to the country's economic development, market competition, social welfare, target populations, and the screen tools adopted [45–47].

Our findings also indicated that the prevalence of depression in China had a wide variation among classifications of the employer, with relatively low prevalence in those employed by Government organizations or State-owned institutions or enterprises and relatively high prevalence in those employed by Private enterprises. Such variation emphasizes the necessity of integrating mental health with employment services, which has not attracted enough attention as occupational physical examination within China. The United Kingdom (UK) has set a good example in regard to this practice. One reflection of the integration of mental health and employment in the UK is the enhanced role of managers in improving workplace mental health [45]. Various organisations in the UK offer mental health training to managers to enhance their mental health awareness and ability to recognize common mental health problems such as depression and anxiety [45]. Considering that mental disorders are particularly frequent but complex and difficult conditions to detect, this practice is vital because it will allow employees with mental health conditions to be identified early before they become clinically ill, and early interventions can be provided to them as well. In the UK, even small and medium-sized enterprises were provided with early and easy access to high-quality, professional advice in response to individual employees’ mental health issues by Occupational Health Advise Services and Fit for Work Service [45]. Other practices from workplaces were also available. For instance, a review of preventing the development of depression in the workplace recommended that cognitive behavioural therapy (CBT)-based interventions (including inoculation training, acceptance and commitment therapy, psycho-education, stress management, and behavioural modification) are effective at reducing universal depression symptom, and exercise-based intervention and team-based participatory intervention are also effective, although they may not as effective as CBT-based interventions [48].

Interestingly, the analysis by industrial classification revealed more possible industry-related factors affecting depression other than the often-known factor like income level. For instance, people who worked in the industry of Real estate had a relatively lower prevalence of depression than those working in the industry of Financial and insurance, even though both industries were considered to have high levels of income. The possible reason is that the Financial and insurance industry is more sensitive to market change; People who worked in the area related to the government, education, and scientific research had a relatively lower prevalence of depression than those working in industries that require manual labour activities (such as Agriculture, Construction, and Mining) and also than those working in industries that involve working face to face with the general public or involve a degree of responsibility coupled with some unpredictability in how their clients might behave towards them (such as Neighborhood service, Wholesale and retail trade, and Accommodation and food service) [49]. The possible reasons are that the former area is not only usually more stable and highly respected because of their relatively higher social status or professions in society, but also have better welfare like higher coverage of health insurance and pension insurance [50]. By contrast, the latter typically either expose workers to high physical risks because of a worse occupational environment [51] or expose workers to high emotional demands and possible adverse social behaviour (like violence and verbal aggression) [49]. The variance by industrial classification indicated that when considering the risk of depression, factors like stability of the position, occupational welfare, occupational environment, emotional demands, and possible adverse social behaviour should be considered in customising industry-related interventions.

Our findings highlighted some high-risk groups for depression, for example, non-employed populations, people who were self-employed or employed by Sole-proprietorship, as well as people who worked in the industries of Agriculture, forestry, animal husbandry and fishing, Neighbourhood service, Construction, and Traffic, storage and mail business. It is worth noting that people's mental health in the above areas or industries got less attention than those in areas or industries like health and scientific research in China [15, 52]. In addition, the above findings are in line with previous studies from other countries to some extent [53–56]. Besides integrating mental health with employment services, more employer- or industry-specific interventions were needed. For non-employed populations, the practice in the UK could also be referenced as a good example. Another reflection of the integration of mental health and employment in the UK is that the health sector has adopted the conclusion that employment is good for mental health and reemployment should be part of any treatment plan for mental disorders [45]. This leads to changes in the outcomes framework of the National Health Service (NHS), which now includes as outcomes employment of people with a mental. Those self-employed or employed by Sole-proprietorship were often overlooked by policy or cannot be reached by the existing system, even in countries (like the UK) with a relatively developed integration of employment and mental health [45, 57–59]. More studies are needed to answer how to improve these groups’ mental health. Nevertheless, enhancing social support by social organisations for these groups of populations may be a beneficial measure [60].

Our findings also highlighted that those employed by unclassifiable employers or working in unclassifiable industries suffered the highest prevalence of depression. These people usually work across employers or industries rather than in a specific or single one, such as the current network game anchors or internet celebrities, who may also promote and sell products at the same time. These people are difficult to be classed to a specific employer or industry traditionally. This characteristic is particularly pronounced in the classification by industry, since a considerable proportion of the people in the other industry classification are self-employed. In recent years, due to the development of the economy and rapid technological progress, the external manifestations of economic activities have become diverse, such as the internet celebrities mentioned above [61]. Those working in these emerging industries not only face enormous opportunities but also suffer from the pressures of intense competition. Some researchers have studied the impact of Internet anchors on the mental health of the public [62, 63], but quite little focus was put on the mental health of people working in these emerging industries, and only some case studies [64]. These areas, which are not easily categorized, also require more attention in future studies.

Our findings indicated that the gender disparity mainly happened among those employed by Sole proprietorship and Private enterprises, as well as those self-employed. The practices mentioned above, including integrating employment and mental health, and enhancing support provided by social organizations, need to pay more attention to females. Unexpectedly, we also found that the industries commonly thought to be female-dominated (like Wholesale and retail trade and Accommodation and food service) had gender disparities that were unfavourable for women. This finding is to some extent contradicting with the evidence from Denmark, where risks of affective- and stress-related disorders were higher for males working in female-dominated areas [65]. The possible reason is that, as discussed above, these female-dominated industries are often emotionally demanding or expose workers to possible adverse social behaviour, because of working face-to-face with the general public or in highly uncertain situations in coping with their clients [49]. In China, the rules or corresponding support for this type of industry have not been perfected compared to the ones in Western countries. On the other hand, we also found that the industries that were commonly male-dominated (like Construction and Information transfer, computer services and software) did not present gender disparities. These findings may imply possible influences of cultural differences. For example, In China, people are mainly influenced by Confucianism and there is a degree of machismo among the man, therefore, in these male-dominated industries, women may be favoured. Nevertheless, we cannot exclude the possibility that in these industries, the unexpected equities in gender may be due to the different division of labour between men and women [66]. For example, in the construction sector, women generally perform relatively low-risk jobs compared to men [66]. The underlying reasons should be explored in future studies, and these anti-common sense findings call for more corresponding interventions.

The primary strength of our study is the availability of data on the employer and industrial classification. Employees in different industries are prone to show agglomeration and heterogeneity. For example, employees in the same industry are likely to face daily (occupational) pressures or challenges that are similar to each other. The evidence-based on the employer and industrial classification contributed to the individualization and pertinence of intervention measures, meanwhile providing policy-makers with the opportunity to balance economic development and improvement of mental health.

Our study was limited by the use of self-reported data, which may be subject to recall bias. Second, the inconsistency of the instrument used in 2016 and 2018. However, a prior validation study showed a strong agreement between CES-D-8 and CES-D-20, with 98% of sensitivity and 83% of specificity [32]. Third, the evidence on the cut-off point for probable depression primarily comes from the western population, whether the validated cut-off point for probable depression meets Asian especially Chinese conditions need more studies in the future. Fourth, people with depression may have been taking antidepressants but without residual symptoms to be identified by the survey instruments. Such people would have been missed by this study, underestimating the proportion of people with depression. Fifth, the economic situation in China is changing rapidly. The results of some industries may be more sensitive to this change, and make their results lack extrapolation. For example, China's control of real estate prices in the past two years may have had an impact on the results of this industry, but how the prevalence of depression in real estate will change? More research is needed. Sixth, we have pooled two surveys from 2016 and 2018 for analysis. As the two surveys were conducted by the same team with the same study design, some individuals will be included in both surveys. In addition, there may be some kinds of inconsistency in social and economic conditions between 2016 and 2018, although, in our experience, there was no obvious market turmoil during this period. We have controlled several covariates (including survey year) as suggested by previous studies which also pooled waves of data for analysis, but the possible bias from this handling was unknown (over or under estimations) [41, 67].

In this study, we identified industries in China where prevalence and gender disparities were relatively higher and where we should focus on in the future plan. Nevertheless, the primary unanswered question of this study is what the contributors to the high prevalence and gender disparities identified in some industries in China. Although some general variables (like age, marital status, education attained, income level, residence place, self-rated health status, and survey year, which we controlled as confounders) were available in the datasets we used, the evidence relating to the associations between these general variables and depression has been extensively documented. In contrast, industry-specific variables such as occupational environment, emotional needs and poor social behaviour, which we mentioned in the above discussion [49, 51], are not available, but these are further key pieces of information for tailored interventions. More research is needed to explore mental health from an industry perspective. In addition, more specific surveys or data with industry-specific information are needed.

## Conclusion

The prevalence of depression in China had a wide variation by classifications of the employer and industrial classification. Gender disparities mainly happened among those employed by Sole proprietorship and Private enterprise, as well as those self-employed, or among those working in industries of Agriculture, forestry, animal husbandry and fishing, Manufacturing, Wholesale and retail trade, and Accommodation and food service. Corresponding evidence can be used for the individualization and pertinence of policies or intervention measures.

## Data Availability

The data are publicly available and can be accessed online (https://opendata.pku.edu.cn/dataverse/CFPS?language=en).
